# Patterns of care analysis for salivary gland cancer: a survey within the German Society of Radiation Oncology (DEGRO) and recommendations for daily practice

**DOI:** 10.1007/s00066-021-01833-x

**Published:** 2021-08-24

**Authors:** Jens von der Grün, Claus Rödel, Sabine Semrau, Panagiotis Balermpas, Daniel Martin, Rainer Fietkau, Marlen Haderlein

**Affiliations:** 1 Department of Radiotherapy and Oncology, University Hospital Frankfurt, Goethe University, Frankfurt am Main, Germany; 2 German Cancer Research Center (DKFZ), Heidelberg, Germany; 3 German Cancer Consortium (DKTK), Partner Site Frankfurt am Main/Mainz, Frankfurt am Main, Germany; 4 Frankfurt Cancer Institute (FCI), Goethe University, Frankfurt am Main, Germany; 5 Department of Radiation Oncology, University Hospital of Erlangen, Friedrich Alexander University of Erlangen-Nuremberg, Erlangen, Germany; 6 Department of Radiation Oncology, University Hospital Zurich, Zurich, Switzerland

**Keywords:** Salivary gland cancer, Molecular diagnostics, Systemic therapy, Radiotherapy, Stereotactic ablative body radiotherapy

## Abstract

**Background:**

Salivary gland cancer (SGC) is rare and a heterogeneous type of cancer. Prospective randomized trials are lacking. No guideline focusing on standard procedures of radiotherapy (RT) in the treatment of SGC exists. Therefore, we surveyed the members of the German Society of Radiation Oncology (DEGRO) to gain information about current therapeutic strategies of SGC.

**Methods:**

An anonymous questionnaire was designed and made available on the online platform *umfrageonline.com*. The corresponding link was sent to all DEGRO members who provided their user data for contact purposes. Alternatively, a PDF printout version was sent. Frequency distributions of responses for each question were calculated. The data were also analyzed by type of institution.

**Results:**

Sixty-seven responses were received, including answers from 21 university departments, 22 non-university institutions, and 24 radiation oncology practices. Six participants reported that their departments (practice: *n* = 5, non-university hospital: *n* = 1) did not treat SGC, and therefore the questionnaire was not completed. Concerning radiation techniques, target volume definition, and concomitant chemotherapy, treatment strategies varied greatly among the participants. Comparing university vs. non-university institutions, university hospitals treat significantly more patients with SGC per year and initiated more molecular pathological diagnostics.

**Conclusion:**

SGC represents a major challenge for clinicians, as reflected by the inhomogeneous survey results regarding diagnostics, RT approaches, and systemic therapy. Future prospective, multicenter clinical trials are warranted to improve and homogenize treatment of SGC and to individualize treatment according to histologic subtypes and risk factors.

**Supplementary Information:**

The online version of this article (10.1007/s00066-021-01833-x) contains supplementary material, which is available to authorized users.

## Introduction

Salivary gland cancer (SGC) is a rare tumor entity including a variety of different histologic subtypes. Due to the lack of prospective, randomized trials, therapeutic strategies remain controversial and no general guideline focusing on detailed recommendations for radiotherapy (RT) in SGC exists. Treatment recommendations are usually based on retrospective data. Surgery, if possible, is the primary treatment of SGC. Furthermore, large retrospective studies indicated a benefit of postoperative external beam radiotherapy (PORT) in locally advanced and/or high-grade SGC [1, 2]. In patients with adenoid cystic carcinomas (AdCC), high linear energy transfer (LET) radiation with protons or carbon ions might be beneficial in case of macroscopic residual or inoperable disease [3]. However, many details on diagnostics and treatment of SGC remain unclear, such as target volume definition, dose prescription, treatment of metastatic disease, and the role of systemic therapy. We thus surveyed the members of the German Society of Radiation Oncology (DEGRO) to gain information about real-life concepts regarding diagnostics and RT for SGC.

## Materials and methods

A pattern of care questionnaire (supplementary table 1) assessing diagnostic and treatment modalities of SGC in radiation oncology departments was developed. The questionnaire focused on general information on participating institutions, indications, diagnostic procedures, target volume definition, RT techniques, and concomitant chemotherapy (CTX). Moreover, five case reports with a total of 18 questions were queried. The questionnaire was reviewed by all listed authors and was made available on the online platform *umfrageonline.com*. The corresponding link was sent to all DEGRO members who provided their user data for contact purposes. Alternatively, a printout PDF copy was sent. The online survey was available from May 5 to August 23, 2020. Within that period, a total of three reminder e‑mails were sent. SPSS (IBM SPSS Statistics, v24.0, Armonk, NY, USA) was used for analysis. Frequency distributions of responses for each question were calculated. The data were further analyzed by type of institution (university department vs. non-university institution/outpatient practice) using Pearson’s chi-squared test. Statistical significance was considered at *p* ≤ 0.05.

## Results

### General information on diagnostics and treatment of SGC

**Fig. 1:**
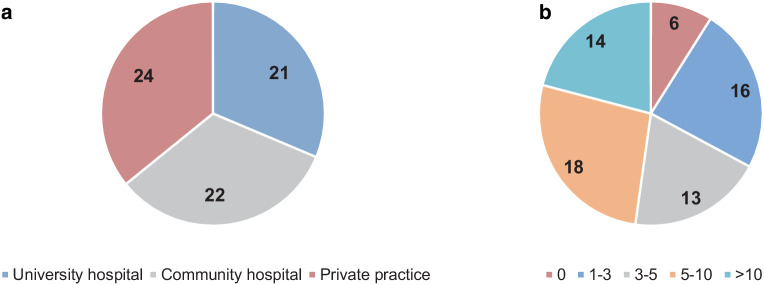
Professional environment of the participants and annual case numbers; **a** professional environment of the participants; **b** distribution of annually treated salivary gland cancer patients reported by participants

Sixty-seven responses were received, including answers from 21 university departments, 22 non-university departments, and 24 radiation oncology practices (Fig. 1a). Six participants reported that their department (practice: *n* = 5, non-university hospital: *n* = 1) did not treat SGC, and therefore the questionnaires were not completed and not considered in the following evaluation. The reported case numbers of the participants are shown in Fig. 1b.

The following annual SGC case numbers were reported (*n* = 61): 26.2% of the participants treat 1–3 patients, 21.3% 3–5 patients, 29.5% 5–10 patients, and 23% >10 patients per year. In 59 (96.7%) of the institutions, head and neck cancer patients are routinely discussed in an interdisciplinary tumor board. A reference pathological second opinion for SGC is gathered in 13 (21.3%) of the participating centers and in 34 (55.7%) of the participating centers, further molecular pathological diagnostics are available (Table 1).

**Table 1:** Numbers of centers routinely using additional molecular pathological diagnostics in salivary gland cancer

Molecular pathological diagnostics	*n*	%
*HER2neu* amplification	33	54.1
Androgen receptor	15	24.6
Estrogen receptor	11	18.0
Progesterone receptor	11	18.0
PD-L1 status	23	37.7
*TRK* fusion	12	19.7
*RET* fusion	6	9.8
Next-generation sequencing/multi gene panels	6	9.8

*PD-L1* programmed death-ligand 1, *TRK* tropomyosin receptor kinase, *RET* ret proto-oncogene

**Fig. 2:**
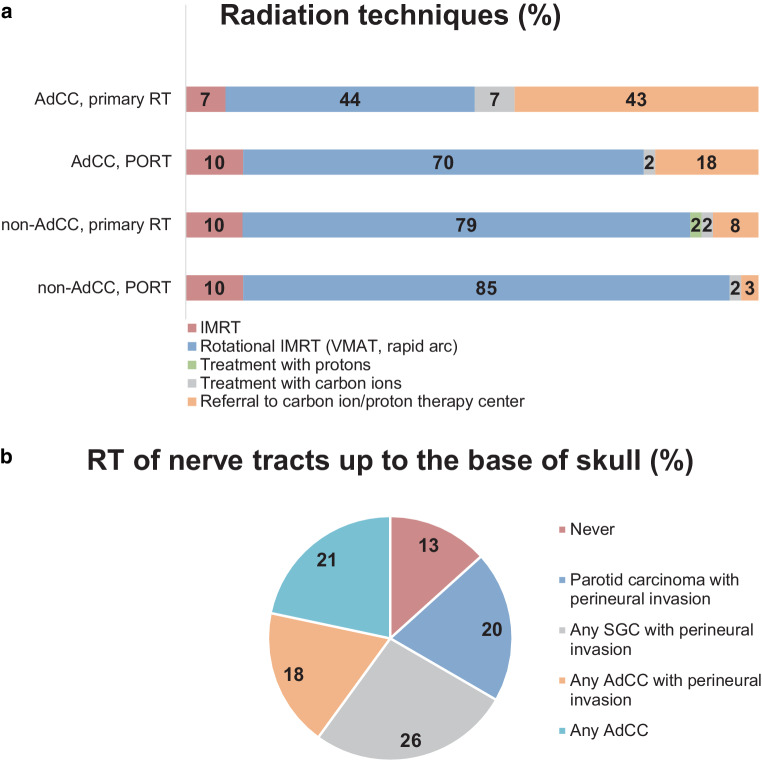
Radiation techniques and indications for nerve tract irradiation. **a** Preferred radiation technique according to tumor subtype and treatment situation, **b** indications for radiotherapy of nerve tracts up to the base of skull. *RT* Radiotherapy, *PORT* postoperative radiotherapy, *IMRT* intensity-modulated radiotherapy, *AdCC* adenoid cystic carcinoma, *VMAT* volumetric arc therapy, *SGC* salivary gland cancer

The most common radiotherapy concept was PORT (*n* = 57, 93.4%), followed by primary (*n* = 3, 4.9%), and palliative RT (*n* = 1, 1.6%). For definitive RT treatment planning, the participants recommended the following imaging modalities: computed tomography (CT) with contrast agent: *n* = 47 (77%); magnetic resonance imaging (MRI) with contrast agent: *n* = 53 (86.9%); fluorodeoxyglucose positron-emission tomography (FDG-PET) CT: *n* = 10 (16.4%); choline/prostate-specific membrane antigen (PSMA) PET-CT: *n* = 1 (1.6%). For PORT treatment planning the following diagnostic procedures were recommended: preoperative CT with contrast agent: *n* = 46 (75.4%); preoperative MRI with contrast agent: *n* = 48 (78.7%); postoperative CT with contrast agent: *n* = 28 (45.9%); postoperative MRI with contrast agent: *n* = 26 (42.6%); preoperative FDG-PET-CT: *n* = 5 (8.2%). The preferred treatment technique as reported by participants was intensity-modulated RT (IMRT). However, the distribution of the preferred treatment technique varies according to tumor subtype (adenoid cystic vs. non-adenoid cystic) and treatment concept (definitive vs. postoperative; Fig. 2a).

RT of the nerve pathways up to the base of the skull is performed as follows: never: *n* = 8 (13.1%); in case of parotid carcinoma with perineural invasion: *n* = 12 (19.7%); in case of all SGC with perineural invasion: *n* = 16 (26.2%); in case of all AdCC with perineural invasion: *n* = 11 (18.0%); and in case of all AdCC: *n* = 13 (21.3%; Fig. 2b).

Of the participants, 21.3% (*n* = 13) never apply concomitant CTX/systemic therapy in patients with SGC in the definitive situation and 39.3% (*n* = 24) in the postoperative situation. Indications for CTX/systemic therapy, when recommended by participants, are listed in Table 2.

**Table 2:** Indications for concomitant chemotherapy in the treatment of salivary gland cancer

Indication for chemotherapy	Definitive treatment situation	Postoperative treatment situation
For all high-risk salivary gland cancers according to recommendations for squamous cell carcinomas of the head and neck region	15 (27%)	10 (24%)
For all kinds of salivary gland cancers according to recommendations for squamous cell carcinomas of the head and neck region	9 (16%)	4 (10%)
According to individual decision	31 (56%)	28 (67%)

Chosen systemic therapy regimes were distributed as follows: platinum-based: *n* = 37; platinum- and 5‑FU: *n* = 11; cetuximab: *n* = 1; targeted therapies: *n* = 1.

### Case studies

**Fig. 3:**
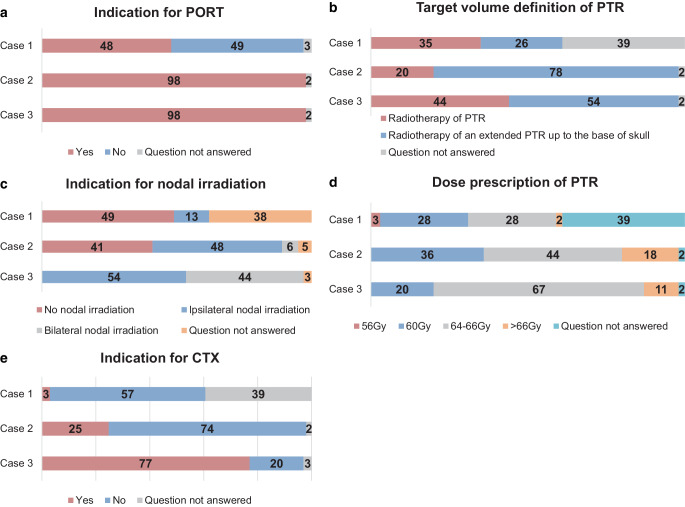
Proposed treatment concepts for cases 1 to 3. **a** Indication for PORT (%); **b** target volume definition of PTR (%); **c** indication for nodal irradiation (%); **d** dose prescription for PTR (%); **e** indication for CTX (%). *PORT* postoperative radiotherapy, *PTR* primary tumor region, *CTX* chemotherapy

*Cases 1 to 3* described two cases of AdCC and one of salivary duct carcinoma (SDC) in the postoperative situation. Detailed case descriptions can be found in supplementary table 1 and results from the survey in Fig. 3. With increasing risk, the number of institutions applying combined chemoradiotherapy (CRT) increases (see Fig. 3e). In case 3, 77% of the participating institutions would apply systemic therapy: 34 institutions would apply simultaneous chemotherapy, 8 institutions simultaneous trastuzumab-based systemic therapy, and 5 institutions trastuzumab-based systemic therapy after radiotherapy.

*Case 4* presented a patient with low-grade acinic cell carcinoma (AciCC) with the following tumor status: pT4 cN0 cM0 R1; second resection not possible or refused by patient. In this case, 55.7% of the participants indicated PORT, 24.6% postoperative chemoradiotherapy (POCRT), 14.8% watch-and-wait, and 4.9% did not answer the question.

**Fig. 4:**
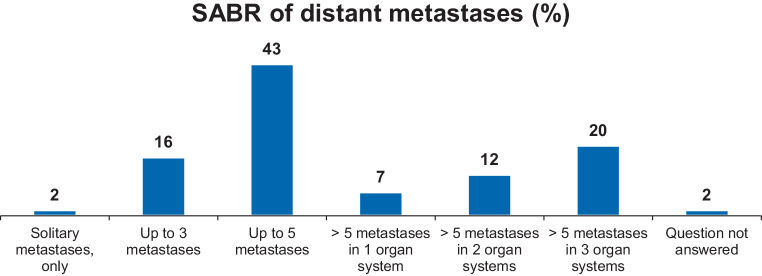
Details on indications for treatment and scope of treatment for stereotactic ablative body radiotherapy of distant metastases. *SABR* stereotactic ablative body radiotherapy

*Case 5* presented a patient with locally controlled AdCC 3 years after primary therapy with first diagnosis of 2 lung and 2 bone metastases. The following treatment options were chosen: 29.5% CTX, 21.3% immunotherapy (IT), 78.7% stereotactic ablative body radiotherapy (SABR), 24.6% surgery, and 4.9% best supportive care (BSC). For details on indications for treatment and scope of treatment for SABR of distant metastases, see Fig. 4.

Comparing university vs. non-university institutions (including community hospitals and private practices), there was a significant difference in the number of patients treated per year and the extent of additional molecular pathological diagnostics. Regarding radiation techniques used, target volume definition, and concomitant CTX application, no significant differences between university and non-university institutions were found (Table 3).

**Table 3:** Comparison of university vs. non-university institutions

Characters	University (*n*)	Non-university (*n*)	*p*-value
**No. of patients per year (*n*** **=** **67)**			**<0.001**
*0*	0	6	
*1–3*	2	14	
*3–5*	1	12	
*5–10*	6	12	
*>10*	12	2	
**Use of further molecular pathological diagnostics**			**0.020**
*Yes*	16	18	
*No*	5	22	
**Radiation technique**			
**Primary RT for AdCC**			0.069
IMRT	2	2	
Rotational IMRT	5	22	
Treatment with protons	0	0	
Treatment with carbon ions	3	1	
Referral to carbon ion/proton therapy center	11	15	
**PORT for AdCC**			0.407
IMRT	3	3	
Rotational IMRT	13	30	
Treatment with protons	0	0	
Treatment with carbon ions	1	0	
Referral to carbon ion/proton therapy center	4	7	
**Primary RT for non-AdCC**			0.133
IMRT	3	3	
Rotational IMRT	13	35	
Treatment with protons	1	0	
Treatment with carbon ions	1	0	
Referral to carbon ion/proton therapy center	3	2	
**PORT for non-AdCC**			0.381
IMRT	3	3	
Rotational IMRT	16	36	
Treatment with protons	0	0	
Treatment with carbon ions	1	0	
Referral to carbon ion/proton therapy center	1	1	
**Use of concomitant CTX for PORT**			0.316
*Yes*	15	33	
*No*	6	7	
**Use of concomitant CTX for primary RT**			0.338
*Yes*	11	26	
*No*	10	14	
**Indications for irradiation of nerve tracts up to the base of skull**			0.560
Never	1	7	
Parotid gland carcinoma with Pn1	6	6	
All SGC with Pn1	6	10	
All AdCC with Pn1	3	8	
Any AdCC	5	8	

*RT* radiotherapy, *PORT* postoperative radiotherapy, *IMRT* intensity-modulated radiotherapy, *AdCC* adenoid cystic carcinoma, *CTX* chemotherapy, *SGC* salivary gland cancer

## Discussion

To the best of our knowledge, the present pattern of care survey for SGC is the first to be carried out among members of the German Society of Radiation Oncologists and among radiation oncologists in general. No prospective trials on RT of SGC have been completed to date. Therefore, we conducted this survey to provide an overview of commonly used diagnostics and treatment modalities applied within the DEGRO. The results are discussed and compared to current literature and guidelines below, with the aim of providing recommendations for practical daily routine.

### Diagnostic imaging for radiotherapy planning

According to current guidelines, salivary gland tumors are locally staged by either contrast-enhanced CT or MRI [4]. Accordingly, the majority of participants recommended initial CT and/or MRI scans (75.8–88.5%). While a prior meta-analysis considered both modalities to be equally effective [5], a more recent meta-analysis by Kong et al. indicated pooled sensitivities and specificities for CT to be 70 and 73% and for MRI to be 80 and 90%, respectively. The authors suggested MRI as the first-choice modality [6]. In addition, 18% of the participants recommended PET-CT for routine tumor staging. Guidelines recommend ^18^F‑FDG-PET-CT for nodal and distant metastases assessment due to its high sensitivity [4, 7]. However, ^18^F‑FDG-PET-CT cannot safely distinguish malignant processes from benign neoplasms such as Whartin’s tumors with high glucose uptake for example [8]. Furthermore, glucose uptake in AdCC is lower than in squamous cell carcinoma (SCC), or may be non-existent [9]. AdCC and SDC often overexpress PSMA (Prostate-specific membrane antigen), indicating a benefit from the use of PSMA PET-CT for these entities [10, 11] rather than ^11^C‑choline PET-CT [10]. For PORT, participants frequently recommended postoperative CT (45.2%) or MRI (43.5%). Indeed, if incomplete tumor resection is expected or pathologically proven, postoperative imaging can guide target volume delineation for adjuvant or additive RT [12]. Recently, an ASCO (American Society of Clinical Oncology) guideline for the management of SGC was published [13]. The authors recommend CT or MRI of the neck as first-choice staging modalities.

#### Take home message

CT scans have an added value when involvement of the bone is suspected and diffusion-weighted MRI scans to rule out perineural invasion and skull base involvement. There is a weak recommendation to use PET-CT in cases of locally advanced or high-grade SGC.

### Dose prescription and target volume delineation

#### Primary tumor region

Early studies regarding definitive photon RT in SGC showed very low locoregional control rates, but the prescribed radiation doses (50 to 60 Gy) were mostly insufficient by current standards [14, 15]. Chen et al. reported improved locoregional control rates when doses were escalated to above 66 Gy [16]. In definitive photon RT of AdCC applying doses >70 Gy, local control rates after 5 and 10 years of 56% and 43% for all T‑stages and of 44% and 30% in T4 tumors only were reported, respectively [17]. According to ASCO guidelines, for patients with unresectable disease, the primary tumor and gross nodal disease should be treated with 70 Gy in 2‑Gy fractions [13]. Regarding the former primary tumor region for PORT, a minimum dose of 60 Gy following complete tumor resection and 66 Gy in case of positive resection margins have been recommended before [1]. Indeed, most participants chose doses between 60 and 66 Gy for PORT. The American guidelines also recommend ≥60 Gy in 2‑Gy fractions to the former primary tumor region in terms of the salivary gland surgical bed and to appropriate nodal levels [13].

##### Take home message

In case of macroscopic disease, a dose of >70 Gy should be applied in the tumor region. In the postoperative situation, dose prescription ranges from 60 to 66 Gy according to risk factors such as microscopic residual disease and/or perineural spread.

#### Elective nodal irradiation

The following recommendations concerning target volume definition in SGC have been proposed:

Clinical target volume (CTV) 1 should include the macroscopic tumor and tumor bed while CTV2 includes CTV1 plus high-risk and ipsilateral nodal levels. If the primary tumor crosses the midline, bilateral nodes should be included [18]. Several guidelines recommend elective nodal irradiation in cN0 patients as well as elective nodal coverage in case of locally advanced T‑stage T3/T4 and in high-grade tumors. However, the histological subtype should be considered. The risk of lymph node metastases is higher in undifferentiated, adeno-, and mucoepidermoid carcinoma compared to AdCC and AciCC [19]. Regarding PORT, recommendations for dose prescription vary between 44, 46, and 50 Gy for the elective CTV2 [4, 13].

##### Take home message

The presence of lymph node metastasis depends on tumor histology, T‑stage, and grading. Elective nodal irradiation should be individually discussed and applied in case of high-grade histology and advanced T‑stage, especially in undifferentiated, adeno-, salivary duct, and mucoepidermoid carcinoma.

#### Radiation of nerve tracts up to the base of skull

The opinions of the participants with respect to target volume definition, especially regarding the inclusion of the nerve tracts up to the base of skull and irradiation of elective neck nodes, varied greatly.

Perineural spread is a common finding in SGC, especially AdCC. In case of perineural spread, the target volume should include the relevant cranial nerve pathways at risk [20]. According to the literature, SGC recurrences to the base of skull were reduced by PORT [13, 21]. Recommended doses for the involved nerve vary from 46 to 54 Gy [13].

##### Take home message

In case of perineural spread, the target volume should include the nerve tracts up to the base of skull.

### Radiation treatment technique

Overall, the preferred treatment technique was IMRT in concordance with the literature [13]. In case of treating AdCC in the primary situation, 50% of the participants would prefer carbon ion RT. Proportions were lower for PORT and non-AdCC. No prospective trials comparing photon with proton or carbon ion radiotherapy exist. Nonetheless, most experience with carbon ion therapy in SGC comes from the treatment of AdCC. A retrospective trial reported superior 5‑year locoregional control in the treatment of AdCC using carbon ion instead of photon RT (60 vs. 40%) [22]. Retrospective long-term data including over 300 patients showed 5‑year locoregional control rates of 58% in all patients and 70.9 and 38.6% in T4a and T4b tumors, respectively [3]. In SGC other than AdCC, the value of carbon ions or protons remains even more unclear. Regarding the combination of IMRT with carbon ion boost, the phase II COSMIC trial [18] enrolled postoperative SGC patients with positive resection margins and/or perineural spread or primarily inoperable patients. Initial results reported an overall locoregional control rate of 81.9% for all patients included and of 89.7% for patients with microscopic incomplete resection margin and/or perineural spread after 3 years. However, local control rates were similar to those reported with photon RT [1, 23].

#### Take home message

According to these data, particle therapy may be used in SGC. Because prospective comparisons with IMRT are scarce, particle therapy remains without clear added value over modern photon therapy so far.

### Concomitant chemoradiotherapy

Twenty-one percent of the participants never recommend CTX for primary RT and nearly 40% for PORT. When CTX is prescribed, it is usually based on an individual decision (60%). Indeed, concomitant RCT is a controversial topic in the treatment of SGC, since hardly any data exist to this regard. A retrospective database analysis could not show any benefit regarding overall survival (OS) for the combination of postoperative RT and CTX [24] and is in line with prior investigations [25]. Unfortunately, high-grade SGC mostly show distant metastases as the first recurrence, which possibly relativizes the potential benefit of an intensified local therapy regarding OS [18, 26]. In matched-pair analyses and small mono-institutional studies, improved locoregional control and/or better progression-free survival in patients receiving platin-based chemotherapy has been reported for SGC [27–29]. Results from a prospective, randomized RTOG 1008 trial (NCT01220583) investigating postoperative radiotherapy with or without weekly cisplatin in high-risk SGC and two other similar trials (NCT02776163, NCT02998385) have not been reported so far. But regarding the fact that radiotherapy leads to high locoregional control rates and most common failures are distant, it has to be assumed that progression-free survival may not be significantly increased in ongoing trials. However, as case reports 1–3 in this study show, simultaneously applied chemotherapy is performed in daily routine in many centers and its implementation correlates with increasing risk factors. Interestingly, in case 3, 13 participants would apply a trastuzumab-based therapy in the postoperative situation. Indeed, some retrospective case series demonstrate improved disease-free survival (DFS) and OS in patients receiving trastuzumab-based therapy or androgen deprivation in the postoperative setting [30, 31]. This targeted therapy may be considered in patients with salivary duct carcinoma with androgen receptor expression and *HER2neu* amplification according to individual risk factors. However, it remains unclear whether these forms of systemic treatment provide any benefit when applied simultaneously to RT and if such combined regimens should be routinely used. According to the new ASCO guideline, concurrent CTX should not be routinely offered outside clinical trials, whether in the definitive or in the postoperative setting [13].

#### Take home message

Results of prospective randomized trials investigating combined CRT in SGC are pending. Concomitant CTX should not be offered routinely.

### Molecular diagnostics

Within this survey, the most commonly performed molecular diagnostics were *HER2neu* amplification, PD-L1 status, and AR status. While the current recommendations for molecular diagnostics with regards to the primary tumor diagnosis are weak, there are indeed several clearer recommendations when systemic therapy is planned: AR in SDC and *NTRK* fusion in MASC; AR, *HER2neu*, and *NTRK* fusion may be offered for non-AdCC; tumor mutational burden (TMB), microsatellite instability (MSI) prior to checkpoint inhibition; next-generation sequencing (NGS) for tumor types with low prevalence. Interestingly, no recommendations regarding the clinical value of the PD-L1 status exist yet [13].

#### Take home message

There is no clear value of comprehensive molecular diagnostics in the primary situation. In case of metastatic disease, molecular diagnostics according to tumor subtype should be performed to evaluate possible targeted therapies. Moreover TMB, MSI, or NGS should be considered.

### Discussion of case presentations

Cases 1 and 2 presented AdCC in different tumor stages following complete resection. AdCC frequently metastasizes, even in cases of early primary tumors. Therefore, controversy exists about PORT for pT1-2N0 tumors to minimize the risk of distant progression, while PORT is strongly recommended for any more advanced tumors [32]. Current guidelines suggest PORT (evidence category 2B) in SGC for intermediate- or high-grade tumors, close or positive margins, perineural invasion, lymph node metastases, lymphovascular invasion, T3‑4 tumors, or any AdCC [4]. However, a National Cancer Database (NCDB) analysis encompassing 1784 AdCC patients found survival differences clearly favoring postoperative irradiation also for the pT1-2N0 subgroup [33]. In contrast, two large and stage-independent analyses from the US American Surveillance, Epidemiology, and End Results Database (SEER) found no benefit for PORT for this cohort [34, 35]. Furthermore, following analysis of the benefit of PORT on local tumor control after adjustment for T‑stage, Ali et al. recommended PORT for all AdCC patients, possibly with the exception of small T1 tumors without adverse features [36]. PORT for all AdCC is also strongly recommended by the current ASCO guidelines [13]. For targeting the primary tumor region, radiation doses of over 60 Gy for AdCC were recommended by Chen et al. [16].

#### Take home message

PORT is recommended for any adenoid cystic carcinoma.

Case 4 presented a T4, low-grade AciCC with positive resection margins. Most participants decided for PORT. As described above, T4 stage and positive resection margins indicate PORT according to the guidelines and a number of authors [4, 37–40]. Since incomplete excisions were associated with impaired survival, PORT should be considered [39]. However, a SEER database analysis including 1241 cases of AciCC from the parotid gland found no survival advantage for early-stage or low-grade tumors and results for highest-stage and highest-grade tumors were inconclusive [41]. Nevertheless, it has to be considered that this analysis was retrospective and without detailed patient characteristics of the two treatment groups (surgery + RT vs. surgery alone). It has to be assumed that patients undergoing radiotherapy had more pathological risk factors. Taken together, giving a clear postoperative treatment recommendation for this scenario remains difficult and individual factors should also be taken into account in a multidisciplinary tumor board.

Case 5 tried to elucidate patterns of care in the oligometastatic situation. Patients with SGC presenting in this disease stage are often treated with individual concepts, mainly due to the lack of effective systemic treatment alternatives and a relatively young age. One of these approaches is metastasis-directed, locally radical therapy via surgery or SABR. Earlier retrospective data demonstrate 5‑year OS rates of 20–54% for the combination of high-intensity metastasis-directed local therapy (i.e. surgery or SABR) and systemic treatment for various entities of oligometastatic head and neck cancer [42, 43]. Advances in RT and especially SABR [44–46] allowed an improvement in disease control and/or survival in different tumor entities in the oligometastatic setting [47–49]. Thus, adding local treatment in this situation appears to be promising. Especially in the case of SABR, this strategy is also based on a strong biological rationale: SABR has well-investigated immunosensitizing features [50]. A large, disease agnostic, randomized trial could recently prove the significant benefit of such an approach for 1–5 metastases when metastasis-directed SABR was added to standard of care for various malignancies [51]. Furthermore, in a subsequent trial by the same group, open for recruitment, this concept is currently being prospectively evaluated even for 4–10 metastases [52]. In the case of SGC, where slowly growing metastases are often observed for some histologic subtypes, metastasis-directed SABR could be a useful tool providing excellent control rates in various organs [53, 54]. A better understanding of the different clinical situations, like oligoprogression and oligopersistence [55], and abandoning the arbitrary oligometastatic definition based solely on the number of metastases, will allow for even better patient selection and oncological results in the future. Moreover, for oligometastatic SGC, the individual histology and tumor grading should also be considered. The current guidelines offer a recommendation in the recurrent and/or metastatic (R/M) setting to evaluate SABR as an option besides systemic therapy. Further, for R/M AdCC and low-grade tumors, SABR may be offered for a limited number of metastases (≤5) [13].

#### Take home message

In oligometastatic disease, SABR and other local therapeutic strategies should be evaluated considering individual histology, progression of disease, and systemic treatment options.

Six participants from private practices and non-university hospitals reported not to treat any SGC at all at their facilities. Depending on histological subtype and the therapeutic setting, up to 43% of the participants would present their patients at a proton/carbon ion tumor center. The treatment of SGC poses a major challenge to any radiation oncologist. Besides the inherent radioresistance of SGC, also the complex shaping of target volumes in combination with their proximity to radiosensitive organs at risk hampers treatment planning [56]. Indeed, all participants would refrain from 3D-RT in favor of more advanced RT techniques as recommended in the literature [56]. Intriguingly, a recent analysis within the NCDB revealed no OS benefit for patients treated at high-volume facilities (HVF) or academic/research institutions. However, patients treated at HVF had more secondary diseases and advanced tumor stages [57]. In order to facilitate the collection of prospective data on the different SGC subtypes and to provide future guidelines, especially considering individual (radiation) treatment options in the different histologic subtypes, the accumulation of patients at certain HVF could be beneficial.

## Conclusion

SGC represents a major challenge for clinicians, as reflected by the inhomogeneous survey results regarding diagnostics, RT, and systemic therapy. The difficulties for practitioners arise mainly from the large number of existing and often rare subtypes with different biological behavior and aggressiveness and the lack of high-level evidence. Future prospective, clinical trials are warranted to improve and homogenize treatment of SGC, and especially to give recommendations for the individual tumor subtypes. Large prospective registers could help to overcome the issues of rarity and heterogeneity of the diagnosis.

## Supplementary Information

Supplementary table 1. Full questionnaire overview.
