# Supplementary material for: Patterns of care analysis for salivary gland cancer: a survey within the German Society of Radiation Oncology (DEGRO) and recommendations for daily practice
Source: Strahlenther Onkol. 2021 Aug 24;198(2):123–34. doi: 10.1007/s00066-021-01833-x (PMC8789700; doi:10.1007/s00066-021-01833-x)
Supplement: Supplementary file 1 — Supplementary table 1. Full questionnaire overview. [file 66_2021_1833_MOESM1_ESM.docx]

| Number | Question / Case description | Anwser |
| --- | --- | --- |
| 1 | Are you employed at an academic hospital, community hospital or private  practice?^#^ | Out-patient practice; Non-university clinic; University clinic |
| 2 | How many cases of salivary gland carcinomas do you treat per year?^#^ | 0; 1-3; 3-5; 5-10; >10 |
| 3 | Are all cases of salivary gland carcinomas at your institution reviewed by a multidisciplinary board?^#^ | Yes; No |
| 4 | Are histopathological diagnoses of salivary gland carcinomas at your institution routinely validated by a reference pathologist?^#^ | Yes; No |
| 5 | Are molecular markers (e.g. Her2neu, TRK fusion) routinely assessed at your institution?^#^ | Yes; No |
| 6 | If so, which markers are routinely assessed?* | Her2neu; Androgen receptor; Progesterone receptor; Estrogen receptor; PD-L1; TRK fusion; RET fusion; Next generation sequencing / Multi gene panels |
| 7 | Which is the most frequent therapy concept for salivary gland carcinoma at your institution?^#^ | Definitive radiotherapy; Postoperative radiotherapy; Palliative radiotherapy |
| 8 | Which imaging, if possible, do you always require for therapy planning definitive radiotherapy?^#^ | FDG-PET-CT; Cholin/PSMA-PET-CT; MRI with contrast medium; CT with contrast medium |
| 9 | Which imaging, if possible, do you always require for therapy planning postoperative radiotherapy?^#^ | Preoperative MRI with contrast medium; Preoperative CT with contrast medium; Postoperative MRI with contrast medium; Postoperative MRI with contrast medium |
| 10 | Which radiotherapeutic technique do you use most frequently for adenoid cystic carcinomas or how do you proceed?^#^ | 3D-RT; IMRT; Rotation-IMRT (VMAT, rapid arc); Tomotherapy; Proton irradiation; Carbon ion irradiation; Patient presentation at a head and neck tumor centre; Patient presentation at a proton/carbon ion tumor centre |
| 11 | Which radiotherapeutic technique do you use most frequently for non-adenoid cystic carcinomas or how do you proceed?^#^ | 3D-RT; IMRT; Rotation-IMRT (VMAT, rapid arc); Tomotherapy; Proton irradiation; Carbon ion irradiation; Patient presentation at a head and neck tumor centre; Patient presentation at a proton/carbon ion tumor centre |
| 12 | Do you apply chemotherapy to patients with salivary gland carcinomas simultaneously with definitive radiation?^#^ | No; Yes, according to the recommendations for squamous cell carcinoma of the head and neck region for all high-risk histologies; Yes, according to the recommendations for squamous cell carcinoma of the head and neck region for all histologies; Yes, according to individual decision |
| 13 | Do you apply chemotherapy to patients with salivary gland carcinomas simultaneously with postoperative radiation?^#^ | No; Yes, according to the recommendations for squamous cell carcinoma of the head and neck region for all high-risk histologies; Yes, according to the recommendations for squamous cell carcinoma of the head and neck region for all histologies; Yes, according to individual decision |
| 14 | If so, which chemotherapies do you most frequently use simultaneously with radiation for salivary gland carcinomas?^#^ | Platinum-based chemotherapy; Platinum- and 5FU-based chemotherapy; 5FU/Capecitabine mono; Taxan-based chemotherapy; Cetuximab; Targeted therapies (e.g. Trastuzumab, androgen blockade); Others |
| 15 | In which cases do you irradiate the nerve pathways to the base of the skull in patients with salivary gland carcinomas?^#^ | Never; In case of parotid carcinoma with perineural infiltration; For all salivary gland carcinomas with perineural infiltration; For all adenoid-cystic carcinomas with perineural infiltration; For all adenoid-cystic carcinomas |
| 16 | *Case 1 description: Adenoid-cystic carcinoma (high-grade) of the left glandula parotis, pT1 pN0(0/15) L0 V0 Pn0 R0 (>5mm) cM0.* |  |
| 17 | Case 1: Postoperative radiation?^#^ | Yes; No |
| 18 | Case 1: If so, extend of the irradiation of the primary tumor region?^#^ | Irradiation of the primary tumor region; Irradiation of the expanded primary tumor region (up to the base of the skull) |
| 19 | Case 1: If so, extend of the irradiation of the lymph drainage areas?^#^ | None; Irradiation of the ipsilateral elective lymph drainage areas; Irradiation of the bilateral elective lymph drainage areas |
| 20 | Case 1: If so, would you apply concomitant chemotherapy?^#^ | Yes; No |
| 21 | Case 1: If so, what dose would you apply to the primary tumor region?^#^ | ca. 56Gy; ca. 60Gy; ca. 64-66Gy; >66Gy |
| 22 | *Case 2 description: Adenoid-cystic carcinoma (high-grade) of the left glandula parotis, pT3 pN0(0/15) L0 V0 Pn1 R0 (>5mm) cM0.* |  |
|  | Case 2: Postoperative radiation?^#^ | Yes; No |
|  | Case 2: If so, extend of the irradiation of the primary tumor region?^#^ | Irradiation of the primary tumor region; Irradiation of the expanded primary tumor region (up to the base of the skull) |
|  | Case 2: If so, extend of the irradiation of the lymph drainage areas?^#^ | None; Irradiation of the ipsilateral elective lymph drainage areas; Irradiation of the bilateral elective lymph drainage areas |
|  | Case 2: If so, would you apply concomitant chemotherapy?^#^ | Yes; No |
|  | Case 2: If so, what dose would you apply to the primary tumor region?^#^ | ca. 56Gy; ca. 60Gy; ca. 64-66Gy; >66Gy |
|  | *Case 3 description: Salivary duct carcinoma of the left submandibular glandula, pT3 pN2b (10/27 with extracapsular extension, 10/17 left, 0/10 right) L1 V1 Pn1 R0 cM0, Her2neu (DAKO 3+).* |  |
|  | Case 3: Postoperative radiation?^#^ | Yes; No |
|  | Case 3: If so, extend of the irradiation of the primary tumor region?^#^ | Irradiation of the primary tumor region; Irradiation of the expanded primary tumor region (up to the base of the skull) |
|  | Case 3: If so, extend of the irradiation of the lymph drainage areas?^#^ | None; Irradiation of the ipsilateral elective lymph drainage areas; Irradiation of the bilateral elective lymph drainage areas |
|  | Case 3: If so, would you apply concomitant chemotherapy/systemic therapy?^#^ | Yes, chemotherapy simultaneously with radiotherapy; Yes, trastuzumab-based simultaneous to radiotherapy; Yes, trastuzumab-based after radiotherapy; No |
|  | Case 3: If so, what dose would you apply to the primary tumor region?^#^ | ca. 56Gy; ca. 60Gy; ca. 64-66Gy; >66Gy |
|  | *Case 4 description: Low-grade acinus cell carcinoma pT4 cN0 cM0 R1, Post resection not possible or rejection by the patient.* |  |
|  | Case 4: Recommendation?^#^ | Postoperative radiotherapy; Postoperative chemoradiotherapy; Aftercare; Systemic therapy |
|  | *Case 5 description: Adenoid-cystic carcinoma, locally controlled, 3 years after primary treatment now first diagnosis of 2 lung metastases and 2 osseous metastases.* |  |
|  | Case 5: Recommendation?^*^ | Chemotherapy; Immunotherapy; Local ablative, stereotactic irradiation; Surgery |
|  | Case 5: If you have chosen a sole, locally ablative, stereotactic approach, how many metastases would you treat in this way? | A solitary; Up to 3; Up to 5; Also more than 5, if technically feasible and located in a maximum of one organ system; Also more than 5, if technically feasible and located in a maximum of 2 different organ systems; Also more than 5, if technically feasible and located in a maximum of 3 different organ systems |

**Supplementary table 1.** Full questionnaire overview.

^#^Single choice question; *Multiple choice question.
